# Congenital Myasthenic Syndrome in Pregnancy: A Case Report and Literature Review on Peripartum Management Strategy

**DOI:** 10.7759/cureus.89337

**Published:** 2025-08-04

**Authors:** Sampath Gnanarathne, Ashani Ratnayake, Dilum Thennewatte, U.A. Isurindi

**Affiliations:** 1 Department of Obstetrics and Gynaecology, Faculty of Medicine, University of Peradeniya, Kandy, LKA; 2 Department of Anaesthesiology and Critical Care, Faculty of Medicine, University of Peradeniya, Kandy, LKA; 3 Department of Anaesthesiology, Teaching Hospital Peradeniya, Kandy, LKA

**Keywords:** congenital myasthenic syndrome (cms), multidisciplinary care team, myasthenic crisis, peripartum management, pregnancy

## Abstract

Congenital myasthenic syndromes (CMS) are a group of rare inherited neuromuscular disorders caused by defects in neuromuscular transmission. Unlike autoimmune myasthenia gravis, CMS typically presents in childhood with variable severity and symptoms, and does not involve autoantibodies. This report presents the management of a primigravida diagnosed with CMS who was generally stable on pyridostigmine therapy but experienced a severe exacerbation during pregnancy. The diagnosis of CMS was made on clinical grounds, but the genetic testing for confirmation of the exact mutation was not available. The patient experienced intermittent mild exacerbations before pregnancy, managed with short courses of prednisolone. A severe exacerbation at 20 weeks of gestation required intensive care unit admission for respiratory support. Despite plans for a vaginal delivery, she developed exhaustion and desaturation during labour, followed by foetal distress, necessitating emergency caesarean section. Postpartum, the patient returned to her baseline functional status, and the neonate showed no signs of neuromuscular weakness.

This case highlights that pregnancy in women with CMS can be safely managed with careful monitoring, multidisciplinary planning, and readiness to escalate care when needed. Delivery plans should be flexible, and management should be tailored to the individual’s clinical course. Since the neonatal outcome is unpredictable in the absence of genetic testing, follow-up care is necessary in all cases.

## Introduction

Neuromuscular junction (NMJ) disorders are a heterogeneous group of conditions that impair signal transmission between motor neurons and skeletal muscle fibres, resulting in fluctuating muscle weakness and fatigability. These disorders are broadly classified based on the site of dysfunction into presynaptic, synaptic cleft, and postsynaptic pathologies [1-3].

Myasthenia gravis (MG) is the most common acquired NMJ disorder, caused by autoantibodies directed against components of the postsynaptic membrane, most commonly the acetylcholine receptor (AChR), but also muscle-specific kinase (MuSK), lipoprotein receptor-related protein 4 (LRP4), or agrin. These antibodies lead to receptor destruction and impaired neuromuscular transmission [1,4].

In contrast, congenital myasthenic syndromes (CMS) are a genetically and mechanistically diverse group of inherited NMJ disorders. They result from mutations affecting presynaptic acetylcholine (ACh) synthesis or release, synaptic cleft ACh metabolism, or postsynaptic receptor clustering and function. Unlike autoimmune MG, CMS are not associated with autoantibodies and does not respond to immunosuppressive therapy or thymectomy [2,4-6].

Understanding the subtype of NMJ disorder is critical, especially during pregnancy and anaesthesia, as it informs therapeutic decisions, monitoring strategies, and anaesthetic planning. There is no universally accepted standard anaesthetic technique for patients with CMS undergoing surgery; thus, the risks and benefits of general and regional anaesthesia must be carefully evaluated on an individual basis. This case report describes the management of a parturient diagnosed with CMS admitted for delivery.

## Case presentation

A 28-year-old primigravida, who was a diagnosed patient of CMS, presented at 40 weeks of gestation for delivery. She had regular follow-up at our hospital, both in the antenatal and neurology clinics. She was diagnosed with CMS at the age of 11 years following an episode of bilateral lower limb weakness. She was then investigated extensively. Findings of her childhood investigations are summarised in Table 1.

**Table 1:** Investigation findings related to myasthenia gravis.

Investigation	Results
Muscle biopsy	Shows more favour of a neurogenic atrophy- spinal muscular atrophy type 3 is a possibility
MRI brain and spine	Normal
Acetylcholine antibody	Negative
Nerve conduction study	Decremental pattern on repetitive stimulation suggestive of a postganglionic neuromuscular junction disorder. The most likely diagnosis is myasthenia gravis. There is no evidence of myopathy

Since the diagnosis, her condition was well controlled with oral pyridostigmine 60 mg six hourly, and she was otherwise medically fit. She developed a myasthenic crisis at 20 weeks of gestation, which required non-invasive ventilation and intensive care admission for seven days. Other than that, her pregnancy was uncomplicated, and her disease condition was managed with the same dose of pyridostigmine during the pregnancy. She was managed by a neurologist throughout the pregnancy.

A multidisciplinary discussion was planned with the involvement of an obstetrician, neurologist, anaesthetist, and neonatologist. It was decided that she should have her delivery at a tertiary care centre where advanced neonatal and intensive care facilities are available. She had a detailed antenatal scan for foetal abnormalities, which showed an adequately grown foetus with no obvious abnormalities. The neonatologist also suggested that the neonate needs to be evaluated immediately after delivery. A normal delivery was planned after multidisciplinary discussion. It was also decided that with the earliest sign of fatigability or respiratory distress, she should be offered an emergency caesarean section. She developed dribbling due to spontaneous rupture of membranes (SROM) on the same day of admission at night and was admitted to the labour room. She had regular contractions for two hours and had 4 cm of cervical dilatation. Epidural analgesia was not provided as the service is not available out of hours. Instead, the patient received intramuscular pethidine and intravenous paracetamol as analgesia. Then the patient became exhausted and complained of difficulty in breathing with tachypnoea (respiratory rate of 22/min). She was afebrile, conscious, and rational, and had a normal haemodynamic parameter. Her saturation on room air dropped to 85% and, therefore, face mask oxygen at 60% was started.

In addition, foetal assessment by cardiotocography (CTG) showed variable deceleration in the early stage of labour. In consideration of foetal distress at the early stage of labour and maternal desaturation, a decision was taken to proceed with emergency caesarean section. Her preoperative investigations, including arterial blood gas analysis, were normal, except for hypocapnia (Table 2).

**Table 2:** Preoperative investigation results. PO2: partial pressure of oxygen; PCO2: partial pressure of carbon dioxide; HCO3: bicarbonate.

Investigation	Parameter	Result
Full blood count	WBC	19.2 × 10^3^/uL
	Haemoglobin	10.4 g/dL
	Platelets	346 × 10^3^/uL
Arterial blood gas	pH	7.39
	PO2	88 mmHg
	PCO2	33 mmHg
	HCO3	20 mmHg
Lactate	1.4	
ECG	Sinus bradycardia	

A lower segment caesarean section (LSCS) was performed under a subarachnoid block with careful positioning of the patient to achieve a sensory level of T4. Supplementary oxygen with a face mask was given to maintain oxygen saturation. Facilities for high-flow nasal oxygen, bilevel positive airway pressure (BiPAP), and intubation with invasive ventilation were prepared and ready to administer in case of further respiratory deterioration. LSCS was uneventful, and the patient was admitted to the ICU for postoperative observation. Pyridostigmine was commenced following admission, and the rest of the supportive care with oxygen continued in the ICU. She remained haemodynamically stable and was off oxygen on the next day.

The baby was monitored by the neonatology team as well as by the neurology team. There was no feeding disability or respiratory difficulties noted. The patient was discharged from the ICU on day two and from the ward on day four.

## Discussion

Unlike myasthenia gravis, CMS is rare and has a diverse presentation. Therefore, there is limited data on the management of CMS in pregnancy [7]. CMS usually presents within the first two years of life, presenting with feeding difficulties and delayed milestones [7,8]. In this patient, the initial diagnosis was made at the age of 11 years, probably due to the fact that her weakness was mainly confined to facial muscles, which would have been easily missed by the carers.

She has been extensively investigated for the cause of weakness at the age of 11 years. Evaluation involved a muscle biopsy as well, in which the myopathy was excluded. According to her past records, the AChR antibody was negative in her serum, and anti-MuSK and anti-LRP4 antibody assays were not done. Several nerve conduction studies confirmed the diagnosis of myasthenia gravis and excluded myopathies. The genetic testing would have confirmed the subtype of CMS, which would be beneficial in genetic counselling of the patient related to pregnancies [7]. The most common pattern of inheritance is autosomal recessive in nature, and, rarely, some subtypes can be autosomal dominant [7]. Therefore, evaluation and follow-up of the neonate is important. Pregnancy may have a worsening effect on the severity of CMS, unlike in myasthenia gravis patients, where there may be a resolution of symptoms due to immunosuppression [9,10].

The most serious maternal complication is a myasthenic crisis, defined as respiratory failure requiring ventilatory support, which can be triggered by infection, fatigue, emotional stress, labour, or medication changes. Our patient experienced a crisis at 20 weeks of gestation, requiring ICU admission, reflecting this risk. Additional concerns include increased sensitivity to respiratory compromise due to physiological reductions in functional residual capacity and increased oxygen demand during pregnancy [2,3]. Some patients can also exhibit cholinergic crisis owing to their treatment. Although our patient did not exhibit cholinergic crisis, she had sinus bradycardia throughout the disease course, which may be due to her treatments.

A study done in England involving 16 women, which assessed 27 pregnancies, revealed that the worsening of symptoms may be seen in 63%, but reversal of baseline functions was seen in all but one patient. The study concluded that it is safe to use the standard medications in pregnancy, assessing their risk according to case by case basis [11].

Another study done on 16 pregnancies of nine patients also revealed variable clinical outcomes; two pregnancies in the same patient showed severe exacerbation of symptoms, and three pregnancies showed mild symptom exacerbations [12]. Out of them, three pregnancies ended up in miscarriages due to uncertain aetiologies. The study concluded that pregnancies in CMS patients are relatively safe and should be managed in specialised centres [12].

There is no standard method of delivery for patients with CMS. As per patients with MG, from an obstetric standpoint, women with CMS are at a higher risk of preterm labour, intrauterine growth restriction, and operative delivery, particularly when maternal muscle weakness compromises the ability to push during the second stage of labour. However, MG or CMS itself are not an indication for caesarean section, and vaginal delivery can be attempted if there is no obstetric contraindication and the patient’s respiratory and neuromuscular status is stable [5].

The optimal mode of delivery in women should be guided by obstetric indications, not by the presence of MG alone. Vaginal delivery is not contraindicated, and many patients can successfully deliver vaginally if neuromuscular and respiratory function are stable [1,2]. However, skeletal muscle fatigue may impair effective maternal expulsive efforts during the second stage of labour, sometimes necessitating operative vaginal delivery with forceps or vacuum assistance [2,3]. In our case, a caesarean was performed in response to intrapartum desaturation and early foetal distress, not solely due to CMS. It is important to recognise that patients with CMS are at increased risk of respiratory decompensation during labour due to reduced ventilatory reserve and increased oxygen demand [7].

Epidural analgesia plays a central role in the intrapartum management of parturients with MG. It provides effective pain relief, reduces oxygen consumption, and limits catecholamine surges that can exacerbate fatigue. Early epidural placement is recommended to minimise stress-related respiratory demand and to facilitate rapid conversion to surgical anaesthesia if operative delivery becomes necessary [4,5]. Care must be taken to avoid excessive local anaesthetic doses, which could lead to motor block and further weaken accessory respiratory muscles. A low-concentration local anaesthetic with opioid admixture is preferred to maintain analgesia without compromising motor function [4].

There is no universally accepted standard anaesthetic technique for patients with CMS undergoing surgery; thus, the risks and benefits of general and regional anaesthesia must be carefully evaluated on an individual basis. General anaesthesia (GA) requires meticulous perioperative planning, particularly due to the unpredictable response of CMS and MG patients to neuromuscular blocking drugs (NMBDs) and anticholinesterase agents. Short-acting agents are preferred to promote early recovery and reduce the risk of respiratory depression [13]. However, residual paralysis after NMBD use may precipitate a myasthenic crisis, while excessive reversal may trigger a cholinergic crisis. Therefore, NMBDs and other agents interfering with neuromuscular transmission should be avoided whenever possible. Alternatively, regional and neuraxial anaesthesia can be considered to avoid the complications associated with GA and reduce the need for sedating or potentially exacerbating medications. Nevertheless, spinal or epidural anaesthesia must be approached with caution, particularly in patients with bulbar or significant respiratory muscle weakness. Mid-thoracic neuraxial blockade may impair accessory muscle function, potentially compromising ventilation. In our patient, spinal anaesthesia was provided to avoid complications associated with GA [13,14]. However, the patient was monitored for respiratory compromise and should have a lower threshold to convert to GA in case of such a complication. Hence, anaesthetic plans for MG patients should be individualised, balancing the benefits of each technique against their potential to precipitate perioperative respiratory compromise.

Postoperative care in parturients with CMS should follow the postoperative management of MG patients and must focus on the early detection of respiratory compromise, continuation of anticholinesterase therapy, and prevention of fatigue-induced exacerbations. Our patient was closely monitored in the intensive care unit due to a mild exacerbation of symptoms during labour.

A critical postoperative priority is the timely continuation of oral pyridostigmine, as interruption or delayed dosing can precipitate neuromuscular weakness. If oral administration is not feasible (e.g., due to nausea, ileus, or nil per oral status), parenteral dosing may be cautiously considered. However, intravenous pyridostigmine is rarely used and must be titrated carefully to avoid cholinergic side effects [4,7].

Analgesia management must avoid respiratory-depressant opioids. A multimodal, opioid-sparing strategy using paracetamol, nonsteroidal anti-inflammatory drugs (if not contraindicated), and local anaesthetic wound infiltration is recommended. Regional techniques, including transversus abdominis plane (TAP) blocks, may also enhance analgesia without compromising respiratory function [1,3].

Neonatal management in the background of maternal CMS requires vigilance for neonatal manifestations. In MG, there is a transient condition caused by the transplacental transfer of maternal AChR antibodies. This occurs in approximately 10-20% of infants born to mothers with autoimmune MG, manifesting as hypotonia, poor feeding, weak cry, and respiratory distress within the first 24 to 48 hours after birth [1,2].

In contrast, infants born to mothers with CMS, which are genetic neuromuscular transmission disorders not mediated by antibodies, as in our patient, do not carry the same risk of neonatal MG. Nonetheless, these neonates may exhibit symptoms related to the specific CMS subtype but typically require individualised neurological evaluation and management [7].

## Conclusions

This case highlights that pregnancy can be safely undertaken in women with CMS with careful multidisciplinary management. Although mild exacerbation of symptoms may occur during labour, outcomes can be favourable with prompt recognition and appropriate obstetric intervention, as seen in this patient who underwent an emergency caesarean section due to foetal distress. The absence of neonatal symptoms and the return of the mother's condition to baseline postpartum further support that CMS does not inherently preclude successful pregnancy outcomes. Management should be individualised, considering the clinical subtype, severity of symptoms, and potential risks to both mother and foetus.
